# *Pleurotus eryngii* Chips—Chemical Characterization and Nutritional Value of an Innovative Healthy Snack

**DOI:** 10.3390/foods12020353

**Published:** 2023-01-11

**Authors:** Charalampia Amerikanou, Dimitra Tagkouli, Thalia Tsiaka, Dimitra Z. Lantzouraki, Sotirios Karavoltsos, Aikaterini Sakellari, Stamatia-Angeliki Kleftaki, Georgios Koutrotsios, Virginia Giannou, Georgios I. Zervakis, Panagiotis Zoumpoulakis, Nick Kalogeropoulos, Andriana C. Kaliora

**Affiliations:** 1 Department of Nutrition and Dietetics, School of Health Science and Education, Harokopio University, 70 El. Venizelou Ave., 17676 Athens, Greece; 2 Department of Food Science and Technology, University of West Attica, Ag. Spyridonos, 12243 Egaleo, Greece; 3 Institute of Chemical Biology, National Hellenic Research Foundation, 48, Vas. Constantinou Ave., 11635 Athens, Greece; 4 Laboratory of Environmental Chemistry, Department of Chemistry, National and Kapodistrian University of Athens, 15784 Athens, Greece; 5 Laboratory of General and Agricultural Microbiology, Department of Crop Science, Agricultural University of Athens, 11855 Athens, Greece; 6 Laboratory of Food Chemistry and Technology, School of Chemical Engineering, National Technical University of Athens, 5 Iroon Polytechniou St., 15780 Athens, Greece

**Keywords:** healthy snack, mushrooms, *Pleurotus eryngii*, Mediterranean, chemical profile, NRF, macronutrients, micronutrients, vitamin D_2_, sensory evaluation, biological value

## Abstract

Nowadays, as the pandemic has reshaped snacking behaviors, and consumers have become more health-conscious, the need for the incorporation of “healthy snacking” in our diets has emerged. Although there is no agreed-upon definition of “healthy snacking”, dietary guidelines refer to snack foods with high nutritional and biological value. The aim of this study was to chemically characterize and determine the nutritional value of an innovative UVB-irradiated and baked snack from *Pleurotus eryngii* mushrooms. *P. eryngii* is an edible mushroom native to the Mediterranean basin. We applied proximate composition, amino acids, fatty acids, vitamins, and macro and trace elements analyses. Also, we computed indices to assess the nutritional quality of food, and we evaluated the sensory characteristics of the mushroom snack. We found high nutritional, consumer, and biological values for the snack. More specifically it was low in calories, high in fibre and protein, low in lipids, without added sugars, and high in ergosterol and beta-glucans. Additionally, it had some vitamins and trace elements in significant quantities. Its NRF9.3 score was considerably high compared to most popular snacks, and the snack exhibited high hypocholesterolemic and low atherogenic and thrombogenic potentials. In conclusion, as a result of UVB-irradiation and baking of *P. eryngii* mushrooms, the snack’s nutritional and biological value were not affected; instead, it provided a “healthy snacking” option.

## 1. Introduction

Currently, the urgent need to address health-related conditions, such as obesity and metabolic disorders, leads to the development and incorporation of ‘healthy’ and ‘functional’ foods in the everyday diet, resulting in a flourishing market of functional foods. Moreover, snack companies are recontextualizing the term “snack food” in light of the consumer’s turn towards modern concepts such as “healthy living” and “active lifestyle” in an effort to reverse their negative dietary reputation by introducing healthier snack products [1,2].

However, the term “healthy snack” still remains unclear, since definitions of “snack” and “snacking” strongly depend on consumers’ perceptions, various socioeconomic data (i.e., age, income, nationality, size of household, etc.) and eating habits, such as food type, frequency of snacking or eating occasions (breakfast, lunch, dinner) [3]. Until now, snacks have been considered to be energy-dense foods that are high in sugar, salt, saturated fats, and calories, and low in nutrients. Nevertheless, their health effects and whether they are beneficial or not have not been fully assessed [2]. Accordingly, the current trend focuses on snacks that disavow the label of “poor-nutrient foods” by incorporating nutrients to enhance health, such as vitamins, amino acids, minerals, fibre, etc. [4].

Nutrient-Rich Foods (NRF) Index scores have been implemented to assess the nutrient profile of the snacks and to determine whether they are nutrient-dense [4]. As no systematic definition or official constituted recommendations of ‘healthy snack’ are currently available, the inventory and tracking of NRF scores of various types of snacks provide essential information regarding their nutritional value. Hence, NRF scores facilitate the formation of more functional and nutrient-dense snack options and the establishment of international consensus nutrition policies and dietary recommendations related to snack consumption [3,5]. Other indices that assess the nutritional quality of the lipid fraction include atherogenicity index (AI), thrombogenicity index (TI) and fatty acids hypocholesterolemic/hypercholesterolemic ratios (h/H) [6,7,8,9].

Since the current trend in the snacks marketplace is the replacement of starchy and fat-rich ingredients (i.e., potatoes, corn, cheese) with healthier ones (i.e., kale, pumpkin, beetroot, grains, broccoli, orange, pineapple, etc.), mushrooms are gaining popularity as alternatives due to their high protein, dietary fibre, copper, zinc, vitamins B, choline, and potassium content, and their low cholesterol content. Additionally, the cultivation of mushrooms is increasing globally, while several health-promoting properties such as their cholesterol-lowering, anticancer, antibacterial, and antidiabetic ones have been well documented [10,11]. A commercially important edible mushroom species is *Pleurotus* which possess a high nutritional value due to protein content and dietary fibre [12]. *Pleurotus eryngii* (*P. eryngii*), mushrooms which are native to Mediterranean regions, are considered a high-quality and low-calorie food, with various pharmacological properties. Their polysaccharides possess several antioxidant, anti-inflammatory, antibacterial, and hypolipidemic characteristics [13].

Thus, the objective of the present work was to chemically characterize and determine the nutritional value of an innovative healthy mushroom-based snack with *P. eryngii* mushrooms that were UVB-irradiated to fortify their vitamin D_2_ content. Therefore, proximate composition, amino acids, fatty acids, ergosterol, vitamins, and trace elements analyses were applied. In addition, nutritional quality indices were calculated to evaluate the nutrient density and healthiness of the produced snack. Finally, the sensory characteristics of the mushroom snack were investigated by a trained sensory panel in order to assess the acceptance of the final product.

## 2. Materials and Methods

### 2.1. Materials

All reagents and chemicals used in this study were of analytical grade and were purchased from Sigma-Aldrich (St. Louis, MO, USA). The EZ:faast Gas Chromatographic Amino Acid Analysis kit was purchased from Phenomenex^®^ (Torrance, CA, USA). Pure gamma-aminobutyric acid (GABA) was purchased from Sigma (St. Louis, MO, USA). β-Glucan Assay Kit (Yeast and Mushroom) was purchased from Megazyme Int. (Leinster, Ireland). Methyl nonadecanoate (98% purity) was obtained from Sigma-Aldrich chemicals (Missouri, USA). ERM^®^- CD281 (elements in rye grass) certified reference material was purchased from the European Commission’s Joint Research Centre (Geel, Belgium). All gas chromatography-mass spectrometry (GC-MS) and inductively coupled plasma mass spectrometry (ICP-MS) solvents were of MS grade and were acquired from Merck (Darmstadt, Germany). All gases were obtained from Linde Hellas (Mandra, Greece). Standards of vitamin D_2_ (ergocalciferol, ≥98%), vitamin A acetate (475,000–650,000 USP units/g) and L-ascorbic acid (≥99%) were purchased from Sigma-Aldrich (Steinheim, Germany). LC and MS grade solvents were acquired from Fisher Scientific (Loughborough, UK), while formic acid (LC-MS grade) was from Scharlab S.L. (Barcelona, Spain).

### 2.2. Methods

#### 2.2.1. Preparation of Mushroom Snack

Whole fresh *P. eryngii* fruiting bodies (caps and stems) cultivated on a wheat straw-based substrate were thin cut (2 mm thickness) with a vegetable processing machine. The sliced mushrooms were seasoned with yeast extract and garlic powder for flavor enrichment at a ratio of 0.5% (*w*/*w*). No artificial preservatives, colors or flavors were added. The slices were then baked in a professional oven at 120 °C for 20 min using no fat or oil. The dry crisps were allowed to cool down, then subjected to UVB-irradiation under a controlled environment of a thermostabilized cabinet to promote vitamin D_2_ concentration levels. The irradiation was applied under the following scheme: linear lamps of 39 W having a definite band of 290–315 nm were placed at a distance of 20 cm from one another; crisps were laid 20 cm far from light source and were UVB treated for 120 min. Six gram portions of the obtained product were packed in sachets made of aluminum foil and sealed airtight. The snacks were stored at room temperature and in darkness until further investigation. *P. eryngii* crisps not exposed to UVB light, and prepared following the aforementioned procedure, were set as replicate control samples to examine the variant of irradiation and the respective vitamin D_2_ content enhancement. Further, microbiological tests were conducted in a certified laboratory to monitor the compliance to safety and hygienic requirements indicated by the relevant legislation for foodstuffs (Regulation 2073/2005). The final snack was prepared and packaged at the facilities of “Dirfis mushrooms” in Euboea, Greece.

#### 2.2.2. Proximate Analysis

##### Crude Protein

Crude protein of the snack was calculated based on its Kjeldahl nitrogen content using the converting factor 6.25. The measurement was done in triplicate.

##### Total Lipid

The lipid content was measured by the colorimetric sulfo-phospho-vanillin reaction, employing commercial sunflower oil as a lipid standard [14,15]. The measurement was carried out in triplicate.

##### Energy Content

The gross energy content of the snack samples was determined with an IKA C4000 (IKA Analysentechnik, Heitersheim, Germany) adiabatic bomb calorimeter. The measurement was done in triplicate.

##### Crude Fibre

Crude fibre was determined by the Weende’s method using a Dosi Fibre apparatus (Selecta, S.A., Barcelona, Spain). The method is based on the solubilization (digestion) of non-cellulosic compounds by a sulfuric acid and potassium hydroxide solution. Crude fibre is the loss on ignition of the dried residue remaining after digestion of the sample, determined gravimetrically.

#### 2.2.3. Free Amino Acids

Free amino acids (FAAs) were extracted from snack as previously described [16]. Three hundred mg of powdered sample were placed into 15 mL screw capped vials. Then we added 5 mL of hydrochloric acid 0.01 M, and samples were magnetically stirred for 15 min at room temperature. Extracts were settled for 45 min, and supernatant (1 mL) was centrifuged at 7200× *g* for 30 min. Supernatants were stored at −40 °C until further analysis.

FAAs profile was obtained with EZ:faast™amino acid derivatization protocol for GC-MS. Solid phase extraction was followed by AAs derivatization and liquid/liquid extraction of the derivatives. One hundred µL of individual extract and 20 nmol of norvaline as internal standards were placed into sample preparation vials. Then, a solid phase extraction was performed via sorbent tips that bind AA derivatives, while allowing interfering compounds to flow through. Derivatization was conducted at room temperature. The derivatized AAs were extracted into 100 µL of isooctane/chloroform, sealed in GC vials and analysed by GC-MS.

A Mass Selective Detector (Electron Impact, 70 eV), a split-splitless injector, and an HP7683 auto sampler were used for the analysis. Derivatized samples (2 µL) were injected into the GC at a split ratio of 1:15. The separation was conducted on a Phenomenex Zebron ZB-A AAs column (length = 10 m, internal diameter = 0.25 mm, film thickness = 25 µm). The carrier gas was high purity helium at a constant flow of 1.1 mL min^−1^. The temperatures of injector and transfer line were at 250 and 340 °C, respectively. Oven temperature was initially set at 110 °C, and then increased to 320 °C at 30 °C/min where it was held for 3 min. A selective ion monitoring (SIM) GC–MS method was used for the detection and quantification of 22 AAs, based on the ± 0.05 RT presence of target and qualifier ions at the predetermined ratios, together with the electronic library “Agilent. L” of the kit. Quantification was performed using norvaline as the internal standard and constructing five points’ reference curves for each AA by standard AAs solutions. GABA was quantitated similarly, by employing pure GABA and extracting the respective m/z (mass to charge ratio) ions. The analysis was done in triplicate.

#### 2.2.4. Fatty Acid Profile

The FAs were determined as fatty acid methyl esters (FAMEs) as described by Alexi et al. [17], after the extraction and direct trans-esterification of the powdered snack in a methanol-toluene (3:2) solution and a freshly prepared acetyl chloride-methanol solution 1:20 (*v*/*v*). FAMEs were analysed by GC-MS (Agilent Technologies, Santa Clara, CA, USA) on an Agilent DB-WAX Column (30 m length, 0.25 mm internal diameter, 0.25-μm film thickness). The oven temperature was initially at 130 °C, raised to 215 °C at a rate of 3 °C/min, then raised to 220 °C at a rate of 0.3 °C/min, and finally, was raised to 240 °C at a rate of 20 °C/min, where it was kept for 12 min. The injector was operated at 25:1 split ratio, and the temperatures of the MS source and quadrupole were set at 230 °C and 150 °C, respectively. Peak identification was based on the retention times and m/z ions of the standard mixture of 37 FAME and was confirmed by means of the NIST05 mass spectra library. FAMEs were semi-quantitated by employing methyl nonadecanoate as the internal standard.

#### 2.2.5. Ergosterol

Ergosterol content was determined according to Sapozhnikova et al. [18] with slight modifications [19]. A 200 mg quantity of sample was saponified with 2 mL of potassium hydroxide (3 M) in methanol under sonication (10 min) and heated (60 °C, 60 min). To avoid the potential photoconversion of ergosterol to vitamin D_2_, all handling was performed under reduced light conditions. The non-saponified fraction was subsequently extracted with 1.5 mL of hexane, and the solvent was evaporated to dryness (Speed Vac, Labconco Corporation, Kansas City, MO, USA). Sterols were derivatized to trimethylsilylethers (TMS) with N,O-bis(trimethylsilyl)trifluoroacetamide (BSTFA) at 70 °C for 20 min, and 1 μL aliquots were injected in the gas chromatographer. An Agilent HP GC 6890 N (Wallborn, Germany) coupled with a mass spectrometer (Agilent HP 5973, Wallborn, Germany) at a split ratio of 5:1 was employed. The analysis of the TMS sterol derivatives was carried out under electron impact ionization (70 eV), and separation was achieved on an Agilent J&W HP-5MS capillary column (30 m × 0.25 mm × 0.25 μm). The carrier gas was high—purity He at a 0.6 mL/min flow rate. The injector and MS detector transfer line were kept at 220 °C and 300 °C, respectively. The oven temperature was set initially at 210 °C, raised to 300 °C at 5.5 °C/min and held for 14 min. The presence of ergosterol was verified by its ion fragments at proper ratios according to literature [20,21]. A 6-point calibration curve, covering the range 0–600 μg was constructed for the quantification of ergosterol; cholesterol was employed as the internal standard.

#### 2.2.6. Macro and Trace Elements

All materials were previously washed thoroughly, soaked in dilute HNO_3_ and rinsed with ultrapure water of 18.2 MΩ cm (Millipore, Bedford, MA, USA). For the preparation of required solutions, class A volumetric glassware was used. Samples were wet digested with the addition of HNO_3_ 65% supra pure and H_2_O_2_ 30% supra pure in a microwave digestion system (Anton Paar Multiwave GO Plus, Graz, Austria) and subsequently diluted to a final volume of 25 mL, according to the procedures described in Grigoriou et al. [22] slightly modified.

Trace elements determination was performed by (ICP-MS), with a Thermo Scientific ICAP Qc (Waltham, MA, USA) instrument. Measurements were performed in a single collision cell mode, with kinetic energy determination (KED) using pure He. Matrix induced signal suppressions and instrumental drift were corrected by internal standardization (^45^Sc, ^103^Rh). Limits of detection (LODs) were calculated by multiplying standard deviation of seven replicate samples prepared at an approximately low concentration by 3.14 [23]. LODs in μg g^−1^ of dry weight were calculated equal to 0.01 for Cd and As, 0.02 for Co and Ni, 0.03 for Pb and Hg, 0.05 for Cr, Cu, Sn, and Mn, 0.16 for Zn, 0.20 for Se, and 0.22 for Fe. Measurement of the macro-elements K, Na, Ca, and Mg in the digested samples was performed by atomic emission spectrometry (AES), using a Varian SpectrAA 200 (Varian, Mulgrave, Australia) instrument.

For quality assurance of the experiment, within each batch of samples we included at least one procedural blank. For the accuracy and precision of the method, a certified reference material (CRM) ERM^®^- CD281 (rye grass) was analysed, and recoveries for As, Cd, Cr, Cu, Fe, Mn, Ni, Pb, and Zn were from 85% to 120%. Recovery tests were performed, through the analysis of metal-spiked samples, and recovery efficiency for spiking sample analysis was ±25% for all elements.

#### 2.2.7. Quantification of β-Glucans

Total and α-glucans were measured according to the instructions of the Megazyme commercial kit ((Megazyme Int., Bray, Ireland), and β-glucans content was calculated by subtraction of α-glucans from the total glucans.

#### 2.2.8. Detection and Quantification of Water- and Fat-Soluble Vitamins

Water soluble vitamins were quantified according to Gentili and Caretti [24], Kivrak et al. [25], and Ciulu et al. [26] while fat soluble A, E, and K were detected and quantified according to Katsa et al. [27] in a Thermo Accela High Pressure Liquid Chromatography (HPLC) equipped with an autosampler (Thermo Scientific, Waltham, MA, USA) and coupled with a Triple Quadrupole Vantage MS (Thermo Scientific, USA) in a private independent laboratory (Eurofins Athens Analysis Laboratories, Athens, Greece).

For the quantification of Vitamin D_2,_ a new LC-MS-MS method was developed. At first, each snack portion out of a total of nine separate samples was treated and analyzed one by one for assessing vitamin D_2_ concentration level. The content of each intact snack package of 6 g was ground to fine powder and mixed to ensure homogeneity before being partly withdrawn for extraction. The extractions were carried out in triplicate per snack sample. Specifically, samples of about 2 g were subjected to a painstaking extraction procedure based on previous studies for the elaborate recovery of vitamin D [28,29]. Pre-weighed samples were hydrolyzed with the prevalence of high pH value conditions and elevated temperature for 45 min. After cooling to room temperature, the solutions were adjusted to approximately neutral pH with the addition of hydrochloric acid. A liquid-liquid extraction with water and n-hexane followed for the formation of a biphasic system, and the collected organic layer was collected after the stabilization of the biphasic for 20 min at ambient temperature. The crude solid extract was recovered using a rotary evaporator operating at 45 °C for the gentle removal of solvents. The dry residue was redissolved in 2 mL of a methanol-water mixture, then centrifuged at 4 °C for 15 min. The supernatant was finally filtered with hydrophobic PTFE filters of 0.45 μm pore size and stored at –20 °C prior to LC-MS injection. The same protocol was followed for the analysis of control samples, i.e., untreated mushroom crisps in relation to UVΒ-irradiation. Vitamin D_2_ was determined by implementing an HPLC tandem mass spectrometric method using an Ultra/HPLC Poroshell HPH-C18 reversed-phase column (particle size 2.7 µm, 2.1 × 150 mm i.d; Agilent, Santa Clara, CA, USA) and a matching guard column. A lipophilic vitamin, namely, vitamin A acetate, was used as an internal standard to correct for differences in the reproducibility of sample treatment and analysis. Experiments were executed in an Agilent 1200 HPLC system equipped with an autosampler (Agilent, Santa Clara, CA, USA), and coupled with a 3200 Q TRAP triple-quadrupole linear ion trap mass spectrometer for mass spectrometric investigation (SCIEX, Framingham, MA, USA). The HPLC methodology applied for separating vitamin D_2_ and internal standard from co-extracted ingredients in the solution for [a period of time]; the analysis was based on a recent work from our research team [30]. Specifically, a binary mobile system compounded from (A) methanol [formic acid 0.1% (*v*/*v*)] and (B) n-hexane [formic acid 0.1% (*v*/*v*)] was used at a constant flow rate of 150 μL/min throughout the gradient solvent scheme. Total run time for each injection was 44 min, embedding 33 min of analysis and the rest of the time for re-equilibration. The autosampler and column operated at approximately 18 °C. Standard solutions, quality control samples, sample solutions, or blanks were injected at a volume of 5 μL. Ionization was achieved via an electrospray ionization (ESI) source operating in positive mode. Three separate multiple reaction monitoring (MRM) transitions were reported for each of the supervised compounds, with precursor and main quantifier ions included. All measurements were performed in triplicate, and reliability of LC-MS/MS based analysis was assured by validating the method. Briefly, selectivity, stability, linearity range, limits of detection and quantitation, and robustness were examined. Pre-spiked matrix-matched standard curves with a concentration range from 2 to 120 μg·mL^−1^ were prepared to include matrix effect and total process recovery for the quantification of vitamin D_2_, exhibiting an adequate degree of linearity as assessed by coefficient of determination (R2 = 0.998). A typical standard curve equation used in our experiments is as follows: Y = 15041X + 82881, where Y stands for the MRM peak area (a.u.) of ergocalciferol MS ion, and X stands for vitamin concentration in the pre-spiked final solution for analysis expressed as μg·mL^−1^. Quality control samples were used to assure accuracy and precision of the analysis for each running batch, ranging within (±)15% of the nominal value. Moreover, the “carry over” effect between injections was investigated to ensure confidence in the analysis. Data acquisition, assessment and processing were performed using Analyst mass spectrometry software (v.1.4.2, SCIEX, Framingham, MA, USA). Statistical calculations were generated with the Origin Pro 8 SR0 (OriginLab, Northampton, MA, USA) statistical software package.

#### 2.2.9. Sensory Analysis

The sensory analysis of the samples was carried out at the Laboratory of Food Chemistry and Technology (School of Chemical Engineering—National Technical University of Athens) by a panel of 12 well trained tasters (4 males and 8 females). The aim was to evaluate the organoleptic properties of four different treatments to which the dried mushrooms were subjected (presented in Table 1). The following sensory indicators were assessed: appearance, odour, taste, texture and aroma/flavor, and all testing was conducted twice for every sample. The tasters were requested to rate the samples for the above indicators with a scale from 1 to 10, (1 minimum and 10 maximum), to hierarchically order them from highest to lowest preference and to give a score from 1 to 10 for their overall impression.

#### 2.2.10. Nutritional Quality Indices

In this study we used NRF9.3 to measure the nutrient density of an innovative snack [31,32]. The NFR9.3 algorithm is based on the sum of nine nutrients to encourage (protein, fibre, vitamin A, vitamin C, vitamin E, calcium, iron, magnesium, and potassium), minus the sum of 3 nutrients to limit (saturated fat, added sugar, and sodium). Daily Reference Values (DRVs) are based on Food and Drug Administration (FDA) standards and are used to compute percentage of daily values of each nutrient.

AI, TI and h/H were computed according to the following equations [8,9]:

AI: [(C12: 0 + (4 × C14: 0) + C16: 0)]/(Σ*MUFA* + Σ*ω*6 + Σ*ω*3)

ΤΙ: (C14: 0 + C16: 0 + C18: 0)/[(0.5 × Σ*MUFA*) + (0.5 × Σ*ω*6 + (3 × Σ*ω*3) + Σ*ω*3/Σ*ω*6]

h/H: (C18: 1*ω*9 + C18: 2*ω*6 + C18: 3*ω*3 + C20: 4*ω*6 + C20: 5*ω*3)/(C14: 0 + C16: 0)

#### 2.2.11. Antioxidant Capacity and Total Phenolic Content

Using the DPPH and FRAP assays, we evaluated the antioxidant potential of the powdered snack methanolic extracts in terms of radical scavenging activity and reducing antioxidant potential, respectively. Results on DPPH and FRAP are expressed as mmol Trolox equivalents per 100 g.

In addition, the Folin-Ciocalteu assay at 750 nm (AnalytikJena, Specord 200 photometer, Jena, Germany) was used to measure the total phenolic content of powdered snack methanolic extracts employing gallic acid as a calibration standard. Results were expressed as mg gallic acid equivalents (mg GAE) per 100 g.

## 3. Results and Discussion

### 3.1. Crude Protein Content

The protein content was found to be 25.43 ± 2.47 g/100 g. This result is in accordance with previous studies performed on mushrooms of the same species [16].

### 3.2. Total Lipids

The total lipid content of the snack was 1.55 ± 0.02 g/100 g.

### 3.3. Crude Fibre

The average crude fibre content was 8.5 ± 0.01 g/100 g.

### 3.4. Energy Content

The energy content was 403.7 ± 2.8 kcal/100 g.

### 3.5. Free Amino Acids

**Figure 1:**
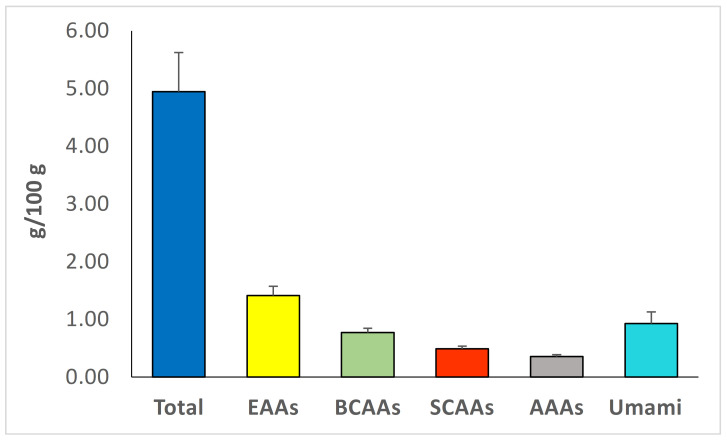
Total and individual groups of free amino acids in the *P. eryngii* mushroom snack. EEAs, Essential amino acids; BCAAs, Branched chain amino acids; SCAAs, Short chain amino acids; AAAs, aromatic amino acids.

The total amount of FAAs was 4.95 ± 0.68 g/100 g, which is in accordance with previous literature concerning *P. eryngii* mushrooms [16,33]. The most abundant FAAs were alanine 0.76 ± 0.04 g/100 g, glutamic acid 0.64 ± 0.09 g/100 g, glutamine 0.49 ± 0.09 g/100 g, leucine 0.42 ± 0.04 g/100 g, and thioproline 0.30 ± 0.05 g/100 g. Essential AAs accounted for 1.41 ± 0.68 g/100 g, branch chained AAs (BCAAs) for 1.41 ± 0.16 g/100 g, short chain AAs for 0.49 ± 0.05 g/100 g, aromatic AAs for 0.35 ± 0.03 g/100 g, and umami taste AAs for 0.93 ± 0.20 g/100 g (Figure 1).

### 3.6. Fatty Acids Methyl Esters (FAMEs)

Fatty acid composition including total saturated fatty acids (SFAs), monounsaturated fatty acids (MUFAs), polyunsaturated fatty acids (PUFAs), n-3 and n-6 fatty acids were detected in the studied snack (Table 2). Up to 16 fatty acids were detected. Linoleic acid (C18:2n6) predominated followed by oleic (C18:1n9) and palmitic (16:0). Unsaturated fatty acids (PUFAs and MUFAs) predominated, representing 82.33% of total FAs, while the SFA were only 15.24%.

### 3.7. Ergosterol

Ergosterol content in the studied snack was 0.55 ± 0.02 mg/g.

### 3.8. Macro and Trace Elements

The concentrations of four macro (calcium, magnesium, sodium, and potassium)—and 13 trace elements (iron, nickel, lead, tin, mercury, cadmium, cobalt, chromium, copper, selenium, manganese, zinc, and arsenic) were recorded. The concentration of K was 24.2 ± 1.8 mg/g and that of Na was 2.1 ± 0.20 mg/g. Mushrooms are foodstuffs characterized by a high-K and low-Na content [34,35]. The rest of the macro elements Mg and Ca were also present at concentrations of 1.6 ± 0.29 mg/g and 0.30 ± 0.20 mg/g, respectively.

Among the trace elements, the most abundant were two essential heavy metals, Fe 460 ± 69 μg/g and Zn 48 ± 6.7 μg/g, followed by Cr 7.2 ± 1.1 μg/g, Mn 4.3 ± 0.39 μg/g and Cu 3.3 ± 0.23 μg/g. The rest of the trace elements were lower than 3 μg/g like Ni 2.8 ± 0.27 μg/g or lower than 1μg/g for Pb 0.24 ± 0.04 μg/g, Se < 0.20 μg/g, Sn 0.07 ± 0.01 μg/g, Co 0.06 ± 0.01 μg/g, Hg 0.05 ± 0.01 μg/g, Cd < 0.01 μg/g, and As < 0.01 μg/g.

### 3.9. Content in Glucans

The content of total glucans was 37.08 ± 1.13% *w*/*w*, of which α-glucans were 4.90 ± 0.27% *w*/*w*, and β-glucans 32.18 ± 0.91% *w*/*w*.

### 3.10. Vitamins Content

The LC-MS/MS analysis revealed a significant ergocalciferol enrichment in the irradiated mushrooms. While the vitamin D_2_ content of non-irradiated *P. eryngii* snack samples was only 0.593 ± 0.073 μg/g (*N* = 5), the irradiated ones presented a more than twelvefold increase of vitamin D_2_ (7.50 ± 0.82 μg vitamin D_2_/g, *N* = 9), as an obvious result of the UVB treatment. The measured D_2_ concentration corresponds to a load of 45.0 ± 4.9 μg vitamin D_2_ in every airtight sealed aluminum foil packet (containing 6 g snack), which equals 1800 international units (IU) [36].

The concentration of other vitamins is presented in Table 3.

ND: non detectable.

### 3.11. Sensory Analysis Results

The results of sensory evaluation are shown in Table 4. Regarding detailed descriptive characteristics, treatment 1 (yeast (0.5%) + taste with the tradename Maxagusto S-99 (1%)) had the highest scoring for most of the appearance characteristics of the respective samples, but scored lowest in odour, crispiness in mouth and taste intensity. Treatment 2 (yeast (0.5%) + taste with the tradename Maxagusto S-99 (1%) + salt (1.5%)) had the highest scoring for key snack sensory characteristics, such as integrity of appearance, easiness of chewing and taste intensity. Treatment 3 (yeast (0.5%) + garlic powder (1%) + salt (1.5%)) had the highest scoring in colour intensity and hardiness in mouth, and Treatment 4 (yeast (0.5%) + taste with the tradename Maxavor Key Beef ΒΧ-H (1%) + salt (1.5%)) in crispiness and aroma/flavor, while at the same time it scored high in bitterness and strange taste. In total, treatment 2 scored higher in overall impression (7.7/10) and 100% of tasters stated that they would consume the respective samples again. Treatments 3 and 4 had significant overall scoring (7.0/10 and 7.2/10 respectively) but samples from treatment 4 were preferable to tasters for consuming them again compared to those from Treatment 3 (71% vs. 43%). On the other hand, the worst treatment method was the first one with 6,6/10 overall impression and only 29% of tasters preferring to consume again the respective samples.

### 3.12. Nutritional Quality Indices

Calculation of NRF9.3 is presented in Table 5. AI was 0.59, TI was 0.31, and h/H was 6.72.

### 3.13. Antioxidant Power and Total Phenolic Content

As demonstrated by the DPPH and FRAP assays, snack extracts exhibited significant antioxidant activity (399.92 ± 10.14 μmol Trolox Equivalents/100 g and 16.31 ± 0.07 μmol Ascorbic Acid Equivalents/100 g, respectively). Total phenolic content was found to be 1.85 ± 0.01 μg GAE/100 g.

## 4. Discussion

During recent years, and especially since the COVID-19 pandemic, while many people have gained weight and adopted unhealthy choices, consumers’ awareness and demand for healthier snack choices have been increasing. Snacks based on mushrooms can provide a healthy alternative as they provide low calories, complex carbohydrates, essential minerals, vitamins, dietary fibre, and are rich in protein, beta-glucans and nutraceutical compounds with antioxidant and anti-inflammatory properties [10]. The aim of this study was to explore whether an innovative mushroom-based snack could be a healthy choice in the human diet. To address this aim, *P. eryngii* mushrooms were UVB-irradiated to enhance vitamin D_2_ content and then baked in a professional oven prior to packaging. The snack was packed in a quantity of 6 g that provides 65% of the recommended daily intake of beta-glucans (3 g) to lower cholesterol levels. Cereal grains are also a source of beta-glucans. We chemically characterized the snack and determined its nutritional value by applying proximate amino acids, fatty acids, ergosterol, vitamins, and trace elements analysis, computing nutritional quality indices and investigating its sensory characteristics.

Overall, our snack could be considered a healthy choice. Regarding its nutritional profile, it has a low caloric content (24.22 kcal/package) and an energy content of 24.22 kcal per serving; the latter is considerably low taking into consideration that most USDA snacks vary between 76 and 214 calories per portion with a mean of 146 ± 25 kcals [37]. Moreover, a 25% protein content is regarded as considerably high for a snack. Higher protein intake in snacks has been associated with several cardiometabolic health parameters in adults (i.e., a negative association with diastolic blood pressure and cardiovascular disease risk), and more protein consumption at more frequent intervals results in higher total and abdominal fat mass loss in an energy balance and deficit intervention in overweight adults [38,39]. Added to the above, the high potassium (24.2 ± 1.8 mg/g) and low sodium (2.1 ± 0.20 mg/g) content suggest that this snack is an extremely healthy food option. In comparison with previous studies, the levels in trace elements were alike [35]. Increased potassium intake lowers blood pressure and contributes to decreasing the risk of heart disease and stroke. On the other hand, intake of too much sodium increases blood pressure and the risk for stroke. The World Health Organisation (WHO) in terms of the five priority actions for non-communicable diseases suggested a global goal of reducing salt intake to less than 5 g (or 2000 mg sodium) per person by 2025 [40].

Regarding fibre content, we measured considerable amounts of crude fibre (8.5 g/100 g) and glucans (37.08 g/100 g) resulting in a total fibre content of 45.58%. Especially in relation to glucans, the snack’s content seems to be unaffected by its preparation (including cooking) process compared with the raw material [19]. This agrees with other studies i.e., in *Shiitake* or *Ganoderma* mushrooms [41,42].

The NRF9.3 score of our snack was found to be 150.89 (or 36.55 per serving), which is considerably high taking into consideration that most popular snacks have NRF scores that vary from −17 to 55. For example, yogurt, milk, and fruit are the most nutrient-dense snacks, while ice cream, pies, cakes and soft drinks are the most nutrient-poor snacks [4]. Of course, it is worth noticing that our mushroom snack is baked and has very low moisture content. Yet, even when compared with other low moisture snacks, such as dry beans, legumes, nuts, and seeds (which have a NRF9.3/100 kcal of 23.1 and a NRF9.3/serving of 44.7), its nutritional quality is high [32].

Sensory evaluation of the different preparation methods of the snack showed that all four treatments had an acceptable sensory quality (>6/10 in overall impression). All samples scored remarkably well in most key snack sensory characteristics, such as integrity of appearance, texture, crispiness, and taste intensity. In total, treatment 2 [(yeast (0.5%) + taste with the tradename Maxagusto S-99 (1%) + salt (1.5%)] scored higher in overall impression (7.7/10), and 100% of tasters would consume samples of this treatment again. Nevertheless, it seems that the preparation method and seasoning do not negatively affect the acceptance of the product, and that a common commercial flavoring along with a low amount of salt could provide a satisfactory outcome.

Regarding FAAs, all the essential FAAs are usually present in mushrooms, comprising 25–40% of total FAAs [43]. In the present work, all the essential FAAs (histidine, isoleucine, leucine, lysine, methionine, phenylalanine, threonine, tryptophan, and valine) were detected in the snack with their total amount reaching 1.41 ± 0.16 g/100 g. Among the essential FAAs identified, leucine predominated, followed by valine. In agreement with our findings, leucine and valine predominated among essential FAAs in *Pleurotus* species cultivated in wheat straw-based substrates [16,44]. BCAAs are necessary for protein synthesis and immunoglobulins, cytokines, and their receptors production. Thus, their availability in the diet is of high importance since they also exhibit a significant immunoregulating role [45]. The total amount of BCAAs in the studied snack was 0.77 ± 0.08 g/100 g, with leucine standing out. Values for free BCAAs content fall within the range (0.064–3.675 g/100 g dw) of the aforementioned in several *Pleurotus* species [16,44,46,47,48,49]. Moreover, the GABA content of the snack (0.03 g/100 g) was within the wide range (0.006 g to 0.390 g/100 g) recorded in previous studies [16,33,47,49]. GABA is a non-protein, four carbon AA present in plants, animals, and microorganisms. It functions as a neurotransmitter of the central nervous system of vertebrates by decreasing neuron activity [50]. In agreement with Tagkouli et al. [16], the concentration of free ornithine was 0.16 ± 0.03 g/100 g.

Our results in fatty acid analysis agree with previous reports for mushrooms of the *Pleurotus* spp [51]. An important finding in this study is the low total lipid and SFA content of the snack (1.55 g/100 g and 0.217 g/100 g, respectively) along with the significant nutritional quality indices of the lipid fraction (AI was 0.59, TI was 0.31, and h/H was 6.72). WHO recommends a reduction of total fat intake to less than 30% of total energy in order to prevent weight gain and risk of noncommunicable diseases and a reduction in saturated fats to less than 10% [52]. The AI, TI and h/H are well known indices of the atherogenic, thrombogenic and hypo- or hypercholesterolemic potential of fatty acids, respectively, and they have been widely used in several foods [53]. According to our scoring, our snack holds a high hypocholesterolemic potential and a low atherogenic and thrombogenic potential compared to other foods. For example, AI in various stages of lactation in milk ranges from 4.08 to 5.13 [54], while eggs have been shown to possess an AI between 0.434 and 0.533, TI between 0.393 and 0.781, and h/H between 1.81 and 2.26 [55]. The aforementioned indices vary in several fish from 0.21–1.41 for AI, 0.14–0.87 for TI, and 0.87–4.83 for h/H [53].

The ergosterol content herein was lower than was reported in previous research [19,56]. Given that ergosterol is the precursor of vitamin D and the mushrooms have been exposed to UV light to increase the vitamin D in the final product, this outcome is valid, indicating the transformation of ergosterol to vitamin D. According to our results, the rapid and straightforward technique of applying ultraviolet irradiation to *P. eryngii* mushrooms proved to be effective at enriching the snack with vitamin D up to 12-fold compared with the non-irradiated control (which was of no nutritional significance regarding the content of the targeted lipophilic vitamin). Recent research results have shown the importance of vitamin D in skeletal and non-skeletal health; serum 25-hydroxyvitamin D levels are not only predictive of bone health but also of cancer and all chronic disease risks [57], while severe or chronic vitamin D deficiency is associated with a number of health risks in certain populations, such as infections [58], cardiovascular diseases, cancer, diabetes [59], hyperparathyroidism, rickets and osteomalacia [60,61]. Consequently, prevention and treatment strategies have been highlighted and reconsidered. In this context, an adequate supplementation with vitamin D has been associated with reduced cancer mortality, e.g., a significant 17% reduction was demonstrated in a study where 2000 IU were administered to adult men and women on a daily basis [62,63,64]. While vitamin D_2_′s effectiveness was considered to be 30% as great as vitamin D_3_, Holick et al. [65] found that vitamin D_2_ was at least as effective in maintaining circulating concentrations of 25-hydroxyvitamin D as vitamin D_3_ [65]. Regular intake of foods fortified with ergocalciferol has been indicated to raise blood levels of its main bioactive metabolite (25-hydroxy-vitamin D) by 1.2 nmol/L for every 40 IU of vitamin, and even up to 48 nmol/L for higher doses following oral administration [66,67]. The content of vitamin D_2_ in our snack falls within the tolerable upper intake level (UL) for vitamin D as stated in the guidelines of health agencies. Specifically, the European Food Safety Authority (EFSA) and the US Food and Nutrition Board of the Institute of Medicine (FNB/IOM) set the UL for adults at 4000 IU per day [67,68,69]. Among the few natural sources containing substantial levels of ergocalciferol, our all-natural and only lightly processed vitamin D-rich *P. eryngii* mushroom product fits the consumers’ expectation for a healthier on-the-go snack. Hence, special alimentation groups such as vegetarians and vegans may incorporate it in their everyday diet. The studied snack may serve to maintain the vitamin D status when consumed as part of a balanced diet and exhibits a strong potential to curb the prevalence of vitamin D deficiency in the adult population.

The UVB-irradiated and baked mushrooms retained only part of the total phenols compared with the phenolic content in *P. eryngii* [56]. Heat treatment has been previously shown to affect the total phenolics and antioxidant activities of *P. eryngii* extracts [70]. Herein, a significant antioxidant capacity was detected in DPPH (399.92 ± 10.14 μmol Trolox Equivalents/100 g) and FRAP (16.31 ± 0.07 μmol Ascorbic Acid Equivalents/100 g) tests on the snack extracts. Islam and his colleagues have evaluated DPPH in a wide variety of edible mushrooms, with a range from 1.36 to 18.56 μmol TE/g with *P. eryngii* having a DPPH of 11.16 ± 0.79 μmol Trolox Equivalents/g [71]. The antioxidant capacity of mushrooms may be influenced not only by the remaining antioxidant content of mushrooms, but also by those derived from baking, the Maillard reaction products.

## 5. Conclusions

The UVB-irradiated and baked snack from *P. eryngii* mushrooms could be considered a healthy snack choice. Based on its low caloric content, its fatty acid profile, but also on its fibre content, potassium and antioxidant potential, this snack should be seen as one that displays cardiovascular protection with a high hypocholesterolemic and a low atherogenic and thrombogenic potential. Its content in vitamin D_2_ is important in terms of covering the pertinent needs of the general population and most prominently of the vegans. The recent trend of European consumers selecting healthier food options and the growing demand for vegan snacks can make the introduction of such an alternative snacking option to the European market a promising one.

## Figures and Tables

**Table 1:** Snack treatments used for the evaluation of the organoleptic properties. Percentage values of ingredients refer to *w*/*w*.

Treatment	Description
**1**	yeast (0.5%) + taste with the tradename Maxagusto S-99 (1%)
**2**	yeast (0.5%) + taste with the tradename Maxagusto S-99 (1%) + salt (1.5%)
**3**	yeast (0.5%) + garlic powder (1%) + salt (1.5%)
**4**	yeast (0.5%) + taste with the tradename Maxavor Key Beef ΒΧ-H (1%) + salt (1.5%)

**Table 2:** Fatty acids identified in the *P. eryngii* mushroom snack as percentage of total fatty acids and as g/100 g of snack.

Fatty Acids		% (*w*/*w*) of Total Fatty Acids		g/100 g of Snack	
Name	Symbol	Mean	SD	Mean	SD
Linoleic	18:2ω6	67.999	0.149	0.969	0.002
Oleic	18:1ω9	12.425	0.240	0.177	0.003
Palmitic	16:0	11.902	0.301	0.170	0.004
Pentadecanoic	15:0	1.637	0.059	0.023	0.001
Stearic	18:0	1.010	0.030	0.014	0.000
Vaccenic	18:1ω7	0.671	0.010	0.010	0.000
Myristic	14:0	0.359	0.013	0.005	0.000
Docosadienoic	20:2ω6	0.212	0.113	0.003	0.002
Margaric	17:0	0.168	0.011	0.002	0.000
Linolenic	18:3ω3	0.163	0.004	0.002	0.000
Palmitoleic	16:1ω7	0.158	0.010	0.002	0.000
Gondoic	20:1ω9	0.148	0.006	0.002	0.000
Behenic	22:0	0.123	0.006	0.002	0.000
Erucic	22:1ω9	0.044	0.003	0.001	0.000
Arachidic	20:0	0.041	0.013	0.001	0.000
Total		97.569	0.977	1.390	0.014
SFA		15.239	0.407	0.217	0.006
MUFA		13.446	0.269	0.192	0.004
PUFA		68.373	0.274	0.975	0.004

**Table 3:** Vitamins concentration in the *P. eryngii* mushroom snack.

Vitamins	Concentration
Vitamin A (μg/kg)	37.4
Vitamin B_1_ (mg/kg)	0.514
Vitamin B_2_ (mg/kg)	9.1
Vitamin B_3_ (mg/kg)	363.0
Vitamin B_5_ (mg/kg)	13,5
Vitamin B_6_ (mg/kg)	1.77
Vitamin B_9_ (mg/kg)	<0.278
Vitamin B_12_ (μg/kg)	<0.45
Vitamin C (mg/100 g)	ND
Vitamin E (mg/kg)	0.398
Vitamin K_1_ (mg/kg)	<0.00179

**Table 4:** Descriptive sensory profiles of the *P. eryngii* mushroom snack as affected by the four different treatments.

Characteristics (1–10, 10 = Maximum)		Treatment			
		1	2	3	4
Appearance	Colour intensity	7.1	7.4	7.7	7.6
	Homogeneity of appearance	7.3	6.8	7.1	7.3
	Homogeneity of shape	6.7	6.7	6.4	6.7
	Visual texture	6.9	6.7	6.7	6.9
	Integrity of samples	7.4	7.4	7.2	7.0
	Defects	2.3	2.0	2.9	1.8
Odour	Odour intensity	5.8	6.1	6.9	6.9
	Defects	1.9	1.5	1.8	1.4
Texture in hand	Hardness	6.1	6.5	6.5	6.7
	Crispiness	7.0	6.6	6.0	7.1
Texture in mouth	Hardness	6.1	6.8	6.9	6.8
	Crispiness	6.4	7.1	6.7	7.5
	Easiness of chewing	7.1	7.6	7.1	7.5
	Defects	1.9	1.6	2.1	1.6
Taste	Taste intensity	6.2	7.4	7.0	7.4
	Saltness	4.6	5.8	5.3	5.8
	Sweetness	4.1	3.9	4.1	4.1
	Bitterness	1.1	1.3	1.1	1.5
	Metallic taste	1.0	1.1	1.1	1.1
	Strange taste	0.9	1.2	1.4	1.5
	Defects	1.7	1.4	1.6	1.5
Aroma/flavor	Aroma/flavor intensity	5.8	7.4	6.8	7.7
	Mushroom aroma/flavor	6.4	6.6	6.6	6.9
	Yeast aroma/flavor	3.0	2.6	2.6	3.1
	Additives aroma/flavor	1.4	2.3	1.8	2.3
	Defects	1.7	1.6	1.6	1.6
After-taste		6.1	6.9	6.6	7.1
Overall impression (1–10, 10 = maximum)		6.6	7.7	7.0	7.2
Order (1 = best, 4 = worst)		3.1	1.6	2.7	2.6
% of tasters who would consume again samples from each treatment		29%	100%	43%	71%

**Table 5:** NRF 9.3 Sample Score Calculation for the *P. eryngii* mushroom snack.

Nutrients	Amount in 100 kcal李of Snack	DRV	PDV	
Protein (g)	8.28	50	17.00	Sum of nutrients to encourage: 153.33
Fibre (g)	11.29	25	45.16	
Vitamin A (IU)	3.09	5000	0.06	
Vitamin C (mg)	-	60	0.00	
Vitamin E [IU (mg)]	0.01	30 (20)	0.05	
Calcium (mg)	7.43	1000	0.74	
Iron (mg)	11.39	18	63.28	
Potassium (mg)	599.43	3500	17.13	
Magnesium (mg)	39.63	400	9.91	
Saturated fat (g)	0.054	20	0.27	Sum of nutrients to limit: 2.44
Added sugar (g)	-	50	0.00	
Sodium (mg)	52.02	2400	2.17	
NRF9.3/100 kcal				150.89
NRF9.3/serving				36.55

DRV: Daily Reference Value; PDV: percent daily value.

## Data Availability

The data presented in this study are available on request from the corresponding author.
